# Prevalence and clinical relevance of extra-cardiac findings at cardiac magnetic resonance imaging

**DOI:** 10.1186/1532-429X-15-S1-P267

**Published:** 2013-01-30

**Authors:** Wieland Staab, Alexander Schwarz, Jan Menke, Judith E Spiro, Paul A Zwaka, Joachim Lotz, Christina Unterberg-Buchwald, Jan M Sohns

**Affiliations:** 1 Radiology, University Medical Center Georg-August-University, Göttingen, Germany; 2 Cardiology and Pneumology, University Medical Center Georg-August-University, Göttingen, Germany

## Background

The aim of this study was to assess the incidence of extra-cardiac findings in patients undergoing clinical cardiac magnetic resonance imaging (CMRI) of the heart including surrounding structures and to determine the influence of those findings on patient's management.

## Methods

N=854 patient studies (median age 58 ± 12 years, male 63%) were included and examined by 1.5 Tesla (T) MR to primarily analyze the cardiac anatomy and secondly the surrounding structures. Extra-cardiac findings were classified as significant (Group A) if they were recommended to additional diagnostics or therapeutical interventions and as non-significant if there was no influence on patient's management (Group B).

## Results

631 patient studies were free of any kind of extra-cardiac pathologies. In the remaining cases, 286 extra-cardiac findings were examined. There were ~0.33 extra-cardiac findings per patient. 49 were defined as significant (Group A) and 237 as nonsignificant findings (Group B). The most common Group A findings were suspicious pulmonary nodules or masses > 4 mm diameter (n=14) and aortic aneurysms (n=5). In Group B, most of the findings were hepatic cysts or hemangiomas (n=50), followed by renal cysts (n=47). 8 malignancies were certainly observed. The most frequent indication for CMRI was evaluation of cardiac stress ischemia (n=501, 59%).

## Conclusions

Extra-cardiac findings in clinical CMRI are common in patients referred to CMRI (26%). Radiologists and cardiologists have to be aware of relevant extra-cardiac findings which might require additional diagnostics or treatment. There is an importance of paying appropriate attention to structures outside of the heart.

## Funding

None.

**Figure 1:**
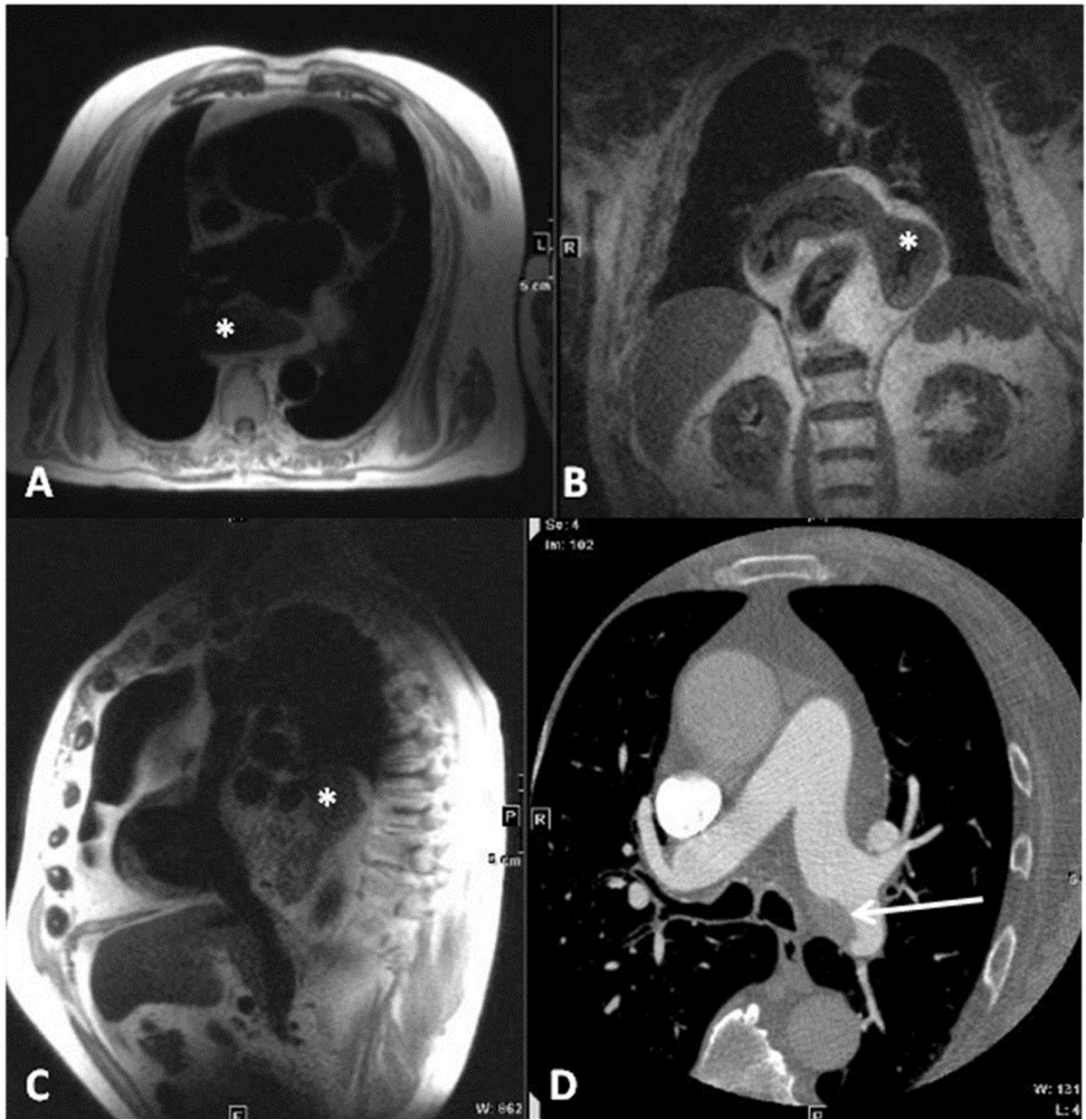
Gastric herniation and angiosarcoma primarily found at CMRI. In a 77 years old male, CMRI primarily detected a thoracic gastric herniation (white stars, A: transversal, Haste, TR 800, TE 46; B: coronal, Haste, TR 800, TE 46; and C: parasagittal, Haste, TR 800, TE 46). An angiosarcoma of the left pulmonary artery was seen in a contrast medium enhanced follow-up thoracic CT scan in the same patient (D: white arrow, axial, 64-slices CT). Recommendation for additional diagnostics was given. Findings were defined as "significant".

**Figure 2:**
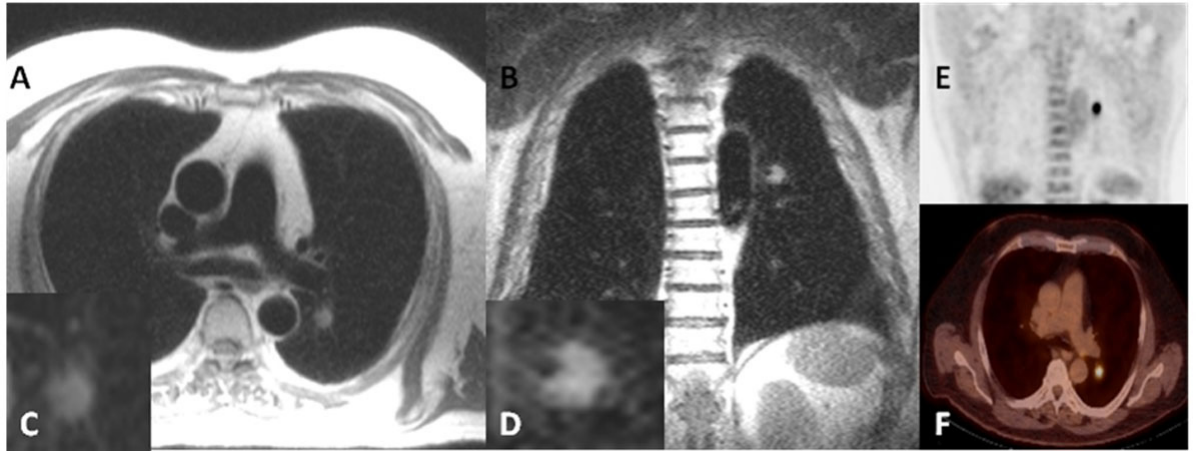
Lung cancer found at CMRI. In a 71 years old patient, CMRI incidentally detected a pulmonary lesion in the left upper lung which was visible on transversal (A: Haste, TR 800, TE 43) and coronal views (B: Haste, TR 650, TE 42). Magnifications are shown in the insets C and D. This "significant" finding was confirmed by a subsequent 18F-FDG PET/CT (E: coronar, F: axial). At histology a bronchial carcinoma was diagnosed and treated. Size 4 x 3 cm.

